# Implementing a smoke-free generation policy for Canada: estimates of the long-term impacts

**DOI:** 10.24095/hpcdp.45.1.03

**Published:** 2025-01

**Authors:** Doug Coyle

**Affiliations:** 1 School of Epidemiology and Public Health, Faculty of Medicine, University of Ottawa, Ottawa, Ontario, Canada

**Keywords:** tobacco smoking, health policy, smokefree generation

## Abstract

**Introduction:**

The aim of this study was to assess the potential impacts of the introduction of a smoke-free generation (SFG) policy in Canada with a perpetual ban on cigarette sales to anyone born after 2009 instigated on 1 January 2025.

**Methods:**

An existing Canadian model relating to smoking cessation was adapted and augmented to assess the impact of an SFG policy on quality-adjusted life years (QALYs), life expectancy, health care costs, smoking-related taxes, and Canadian tobacco industry gross domestic product (GDP). The cumulative impact of the policy for the entire Canadian population was assessed for time horizons up to 90 years with an annual discount rate of 1.5%.

**Results:**

After 50 years, this SFG policy would lead to 476814 more QALYs, $2.3 billion less in health care costs, $7.4 billion less in smoking-related taxes and a $3.1 billion reduction in tobacco industry GDP. The combined value of health benefits gained and health care costs averted would exceed the sum of tax revenues foregone and reduced GDP, if the value of a QALY was at least $17147. Use of higher discount rates and inclusion of unrelated health care costs had little impact on the interpretation of the results.

**Conclusion:**

The implementation of an SFG policy will bring substantive health benefits to the population in Canada. Although health care cost savings are lower than the combination of lost tax revenues and the decline in the GDP from the Canadian tobacco industry, the value of the health benefits realized outweigh the negative offsets.

**Highlights**

- A smoke-free generation (SFG) policy involves prohibiting the sale of tobacco products to people born after a specific date for their lifetime.
- The impact of an SFG policy on quality-adjusted life years, life expectancy, health care costs, smokingrelated taxes and Canadian tobacco industry gross domestic product was assessed for up to 90 years.
- Implementing an SFG policy leads to substantive health gains and significant health care cost savings.

## Introduction

Although the prevalence of smoking has decreased consistently over the last 50years, the proportion of Canadians who are current daily smokers showed a small increase, from 8.4% to 9.1%, in 2022.^1^ Despite the decreasing trend in prevalence, smoking remains a major cause of preventable disease and mortality in Canada.^2^

The current target of federal government policy is to reduce tobacco use to less than 5% by 2035.^3^ The strategy focuses on helping Canadians who are current smokers to quit and on protecting those who do not smoke, particularly youth, from developing a tobacco addiction. However, provinces vary with respect to smoking cessation initiatives such as the age when it becomes legal to purchase tobacco (between 18 and 21 years).

Despite the restrictions on the sale of tobacco products, responses to the Canadian Student Tobacco, Alcohol and Drugs Survey show that 3% of students in Grades 7 to 12 in 2018 to 2019 (aged 11–18 years) were currently smoking cigarettes.^4^ In addition, 58% of the students responded that it would be “fairly easy” or “very easy” to get a cigarette if they wanted one.^4^ By the time they were aged 18 years, 7.5% of males and 4.6% of females reported that they were current daily smokers.^4^

Various jurisdictions across the world have contemplated introducing a smoke-free generation policy (SFG) to limit tobacco consumption.^5^,^6^ An SFG policy involves prohibiting the sale of tobacco products to people born after a specific date for their lifetime. Concerns with current legislation are that smoking initiation continues among individuals who are prohibited from purchasing tobacco and that those individuals for whom the protective measures apply will reach an age where the restriction of tobacco sales is no longer valid. An SFG policy can address both of these concerns.

To determine the impacts of an SFG policy, an existing Markov model for assessing the impact of smoking cessation in Canada was adapted to determine the impacts on life expectancy, quality-adjusted life years (QALYs), smoking-related health care costs, taxes raised through tobacco sales and the Canadian gross domestic product (GDP).

## Methods

***Analytical approach***

An approach was adopted that was similar to a previous analysis that examined the impact of vaping by teenagers on the uptake of smoking tobacco and the subsequent impacts on QALYs and costs.^7^ The revised model adopts the same approach to modelling smoking commencement, cessation and relapse and focuses on health states related to smoking status.^7^ The model simulates a Canadian population aged 15 years and older and the transition of individuals between the following states: current smokers, former smokers, never smokers and dead. The model predicts the proportion of the cohort in each of these states every 6 months. The numbers of males and females turning age 15 years in 2025 were based on the most recent census figures adjusted for both mortality and immigration.^8^-^10^

All data used in the analysis are presented in Table 1.

**Table 1:** Comprehensive data for Markov model stratified by gender—transition probabilities, disease prevalence, relative risks, costs and utility values

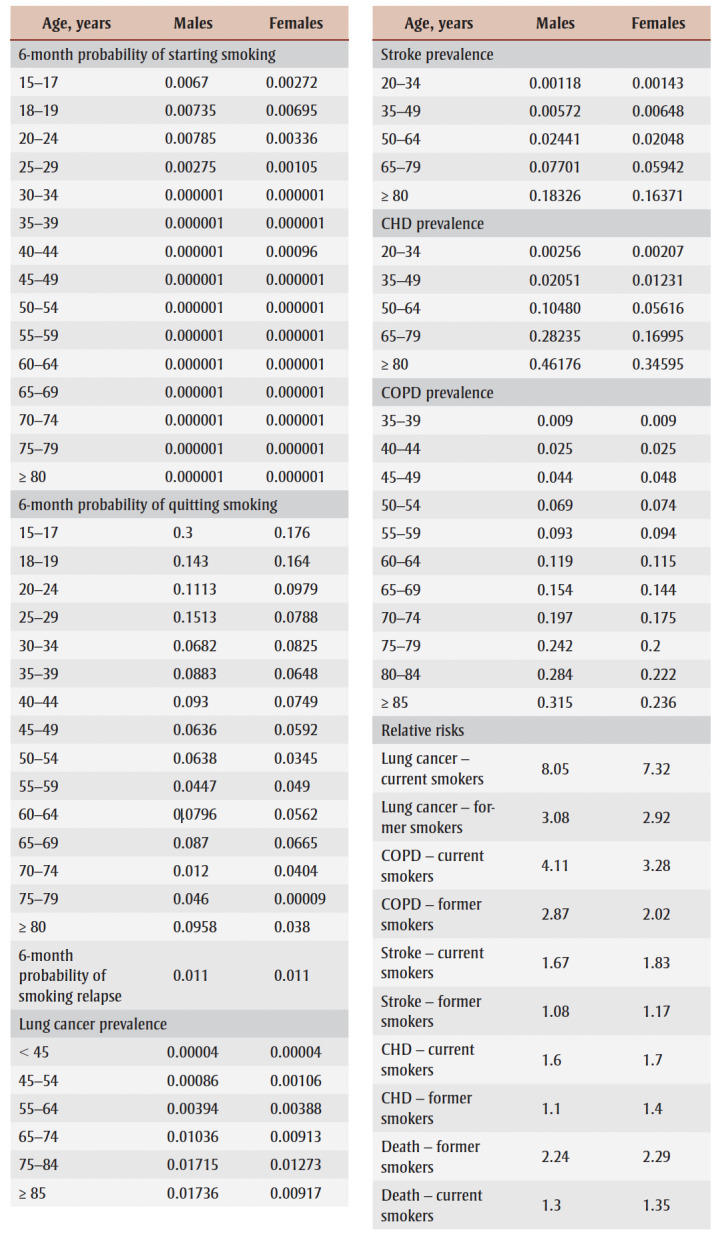 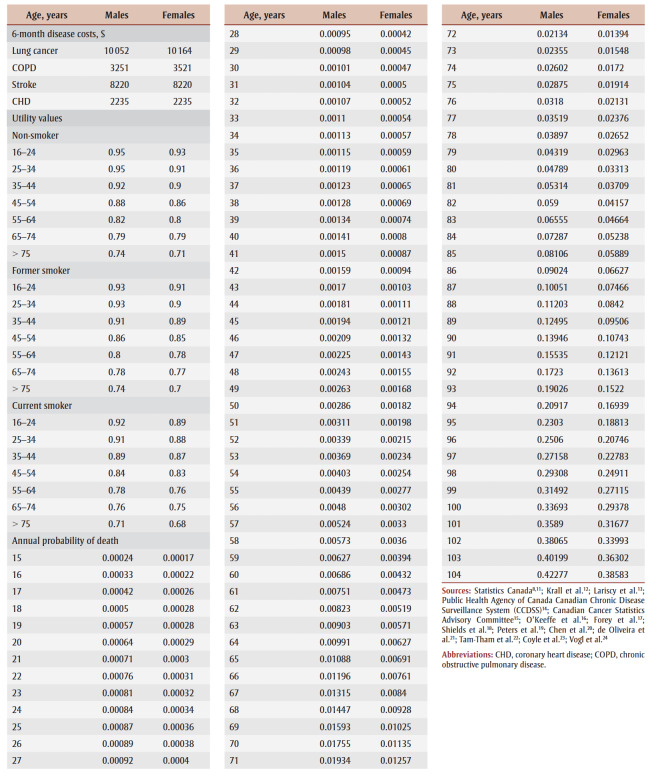

***Comparators***

Two scenarios were compared: (1) the status quo, where no SFG policy is implemented and the uptake and retention of smoking behaviour over the course of the model is informed by current age–gender specific start and quit rates; and (2) the existence of an SFG policy whereby a perpetual ban is instigated, on 1 January 2025, on cigarette sales to anyone born after 2009, with the aim of no uptake of smoking by Canadians born after 2009.

Despite current age restrictions on the purchase of tobacco, consumption appears to begin during the early teenage years. To allow for potential access to tobacco for those in their early teens during the initial years of the SFG policy, two scenario analyses were conducted: assuming a 5‑year lag such that those born between 2009 and 2013 could obtain tobacco illegally despite the restrictions of an SFG policy from 1 January 2025; and assuming an SFG policy that reduces the uptake of smoking in the relevant age cohorts by 90% rather than 100%.

***Model design***

**Figure 1:**
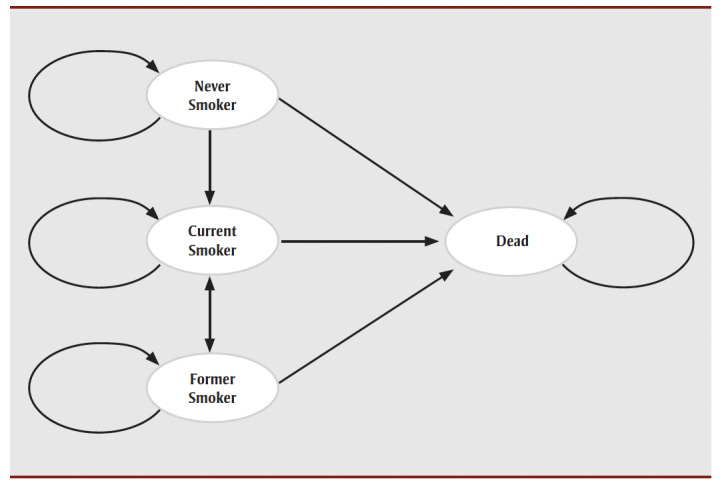
Schematic showing the Markov model for transition of a population cohort between states of being a current smoker, former smoker, never smoker or dead from age 15 years to death

Markov models for both males and females were developed to model the transition of a population cohort between states of being a current smoker, former smoker, never smoker or dead from age 15 years to death (Figure 1). Stratification by gender was necessary due to differential input parameters relating to onset of smoking, smoking cessation, utility values and underlying mortality data. The model facilitates estimating the cumulative impacts on life expectancy, QALYs, health care costs, tax revenue and GDP over time horizons of up to 90 years. 

***Transitions***

For the no SFG policy scenario, age–gender specific probabilities were required for the transition from never smoker to current smoker (“start”), current smoker to former smoker (“quit”) and former smoker to current smoker (“relapse”). Probabilities of starting and quitting were derived from data from the 2017 Canadian Tobacco, Alcohol and Drugs Survey (CTADS),^11^ while the long-term probability of relapse after cessation was based on Krall et al.^12^ For the SFG policy scenario, in the base (primary) analysis, after policy initiation all individuals were assumed to remain in the never smoker category until death.

The six-month probabilities of dying for each age–gender cohort by smoking status were derived through a calibration process using Statistics Canada mortality data, current smoking status from CTADS data and the relative risk of mortality by smoking status.^13^

***Costs of smoking-related diseases***

The base analysis focuses on the health care costs associated with the four smoking-related diseases that comprise 75% of smoking-related mortality in developed countries: chronic obstructive pulmonary disease (COPD), coronary heart disease (CHD), stroke and lung cancer.^25^ The six-month probabilities of having each of these diseases for each age–gender cohort by smoking status was derived through a calibration process using prevalence data, current smoking status data and the relative risk of disease by smoking status. Age–gender specific prevalence rates were obtained from Canadian population-based data for COPD, CHD and stroke.^14^ Due to the lack of prevalence data, lung cancer prevalence was estimated by dividing available incidence data by estimates of average life expectancy.^15^ Relative risks of disease by smoking status were obtained from published literature.^16^-^19^

Average six-month Canadian costs for each of the diseases were derived from the available literature.^20^-^23^ Costs were estimated in 2023 Canadian dollars, with adjustments where necessary using the Bank of Canada Inflation Calculator.^26^

***Additional health care costs***

Delaying mortality and avoiding smoking-related diseases may lead to an increase in health care expenditures for other conditions. Thus, a scenario analysis was conducted to include health care costs that are not related to the smoking-related diseases. Values for six-month additional health care costs were derived for individuals with differential values applied depending on whether the individuals died or survived the cycle.^27^,^28^ These values were obtained by adjusting available estimates of annual health care costs by age group and gender, and health care costs in the last year of life by the prevalence of smoking-related diseases and their costs and by the age–gender specific mortality rates.

***Utility values***

Utility values represent individuals’ preferences for different health states on a scale of 0 to 1 where 0 represents death and 1 represents perfect health. Age–gender specific utility values by smoking status were obtained from Vogl et al.^24^ Values incorporated the impact of smoking-related diseases on health-related quality of life. Thus, further disutilities associated with the four smoking-related illnesses were excluded as this would lead to double counting of the impact of smoking.

***Tobacco industry GDP***

Tobacco industry GDP is a measure of the total output created through the production of tobacco-related goods and services. The current magnitude of the GDP for the Canadian tobacco industry was divided by the estimated number of smokers in Canada to obtain an average annual contribution to the GDP per smoker of $711.25.^29^

***Tobacco-related taxes***

The average tobacco-related tax contributed by a smoker in Canada was estimated at $1685 a year.^30^

***Analysis***

The model calculated the impact of enacting an SFG policy for each year of the analysis across all affected birth year cohorts. For Year 1 (2025), outcomes were assessed for those born in 2010 (i.e. for the year they turned 15). For Year 2 (2026), outcomes were assessed for both those born in 2010 (i.e. for the year they turned 16) and those born in 2011 (i.e. for the year they turned 15). Calculations for further years followed the same logical approach.

## Results

The cumulative impact each year up to 90 years, and specifically for time horizons of 10, 25, 50 and 90 years are shown in Figures 2a, b and c and Tables 2, 3a, 3b, 3c and 3d. Analysis determined the threshold value of a QALY whereby the value of health benefits (QALYs weighted by the threshold value) plus health care costs avoided exceeded the sum of tax revenues foregone and the decline in GDP.

**Figure 2A:**
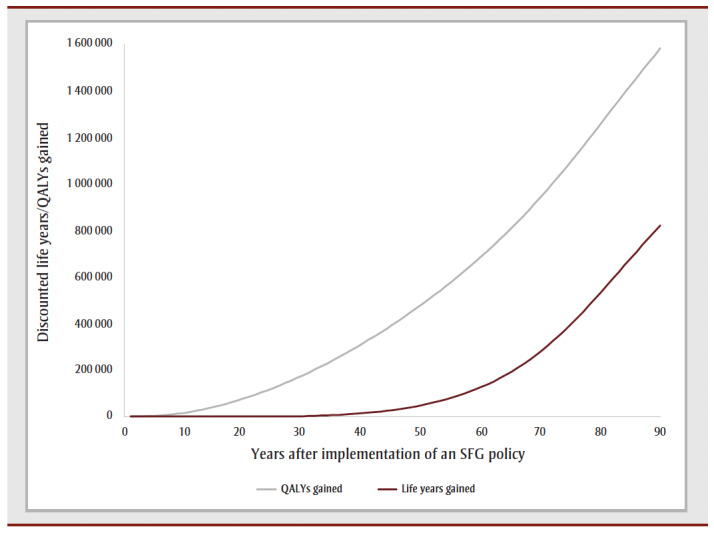
Cumulative outcomes up to 90 years—QALYs and life years gained

**Figure 2B:**
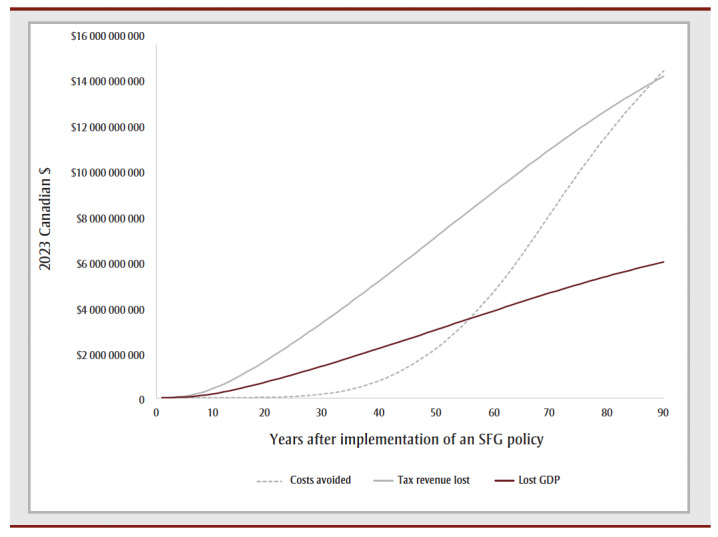
Cumulative outcomes up to 90 years—financial gains and losses

**Figure 2C:**
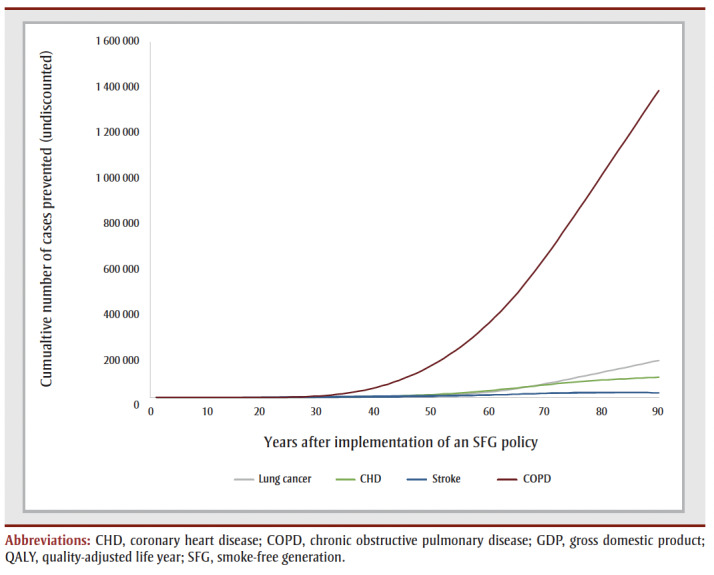
Cumulative outcomes up to 90 years—number of cases prevented

**Table 2:** Base results of the impact of introduction an SFG policy over time—life years, QALYs, health care costs, tax revenue from smoking, GDP and incident cases of smoking-related diseases

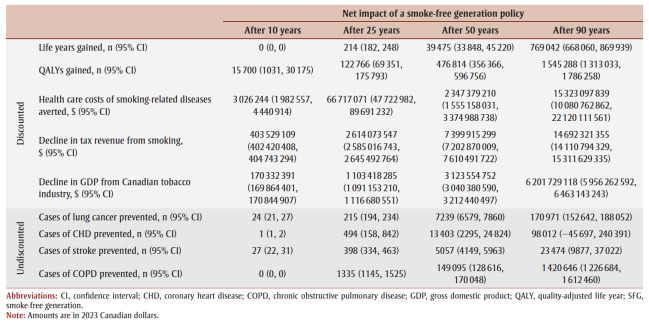

**Table 3A:** Disaggregated results for life years, QALYs, health care costs, tax revenue from smoking, GDP and incident cases of smoking-related diseases in the absence and presence of an SFG policy and net impact after 10 years

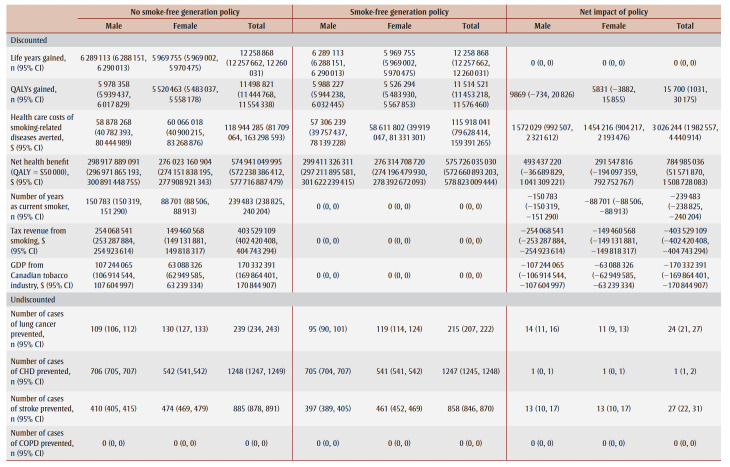

**Table 3B:** Disaggregated results for life years, QALYs, health care costs, tax revenue from smoking, GDP and incident cases of smoking-related diseases in the absence and presence of an SFG policy and net impact after 25 years

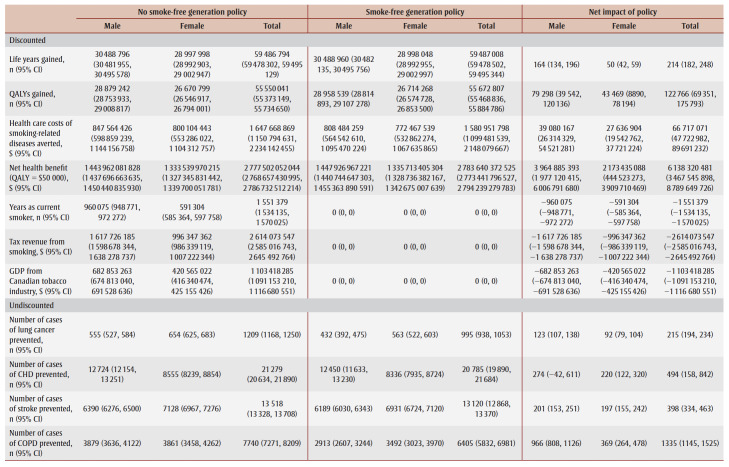

**Table 3C:** Disaggregated results for life years, QALYs, health care costs, tax revenue from smoking, GDP and incident cases of smoking-related diseases in the absence and presence of an SFG policy and net impact after 50 years

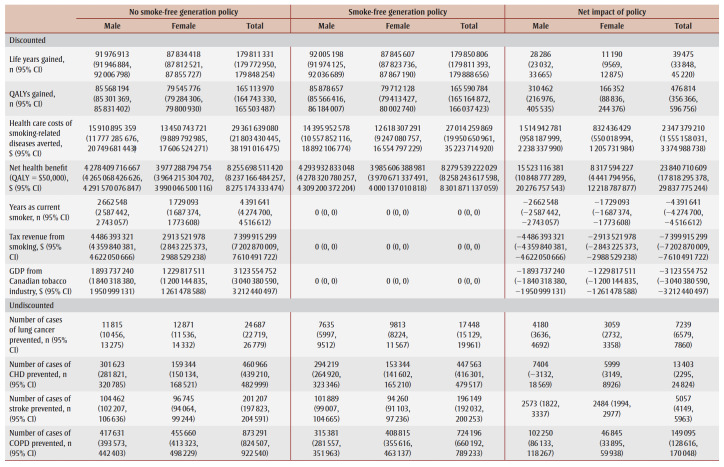

**Table 3D:** Disaggregated results for life years, QALYs, health care costs, tax revenue from smoking, GDP and incident cases of smoking-related diseases in the absence and presence of an SFG policy and net impact after 90 years

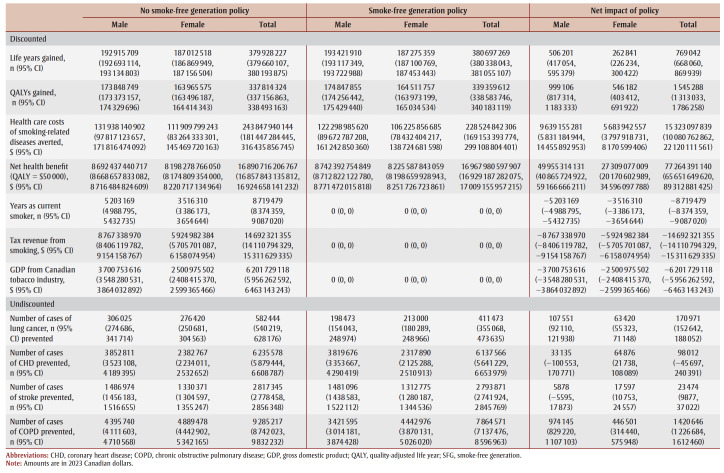

Analysis adhered to guidelines for economic evaluations in Canada.^31^ To account for uncertainty in inputs, outcomes were assessed by probabilistic analysis through a Monte Carlo simulation of 5000 replications to ensure stability of the data. Probability distributions were used to account for uncertainty around the parameters of interest, and the choice of distribution was based on common practice.^31^ To allow for society’s preferences with regards to the timing of events, an annual discount rate of 1.5% was applied to all costs and utilities.^31^

***Scenario analyses***

The following scenario analyses were presented as cumulative impacts at 50 years:

- A 90% reduction in smoking initiation to allow for potential illicit market adoption.
- An SFG policy only impacting initiation of smoking after 5 years based on the assumption that individuals may be able to access cigarettes despite the restrictions.
- Different discount rates (0%, 3% and 5%) to assess the impact of discounting.
- Inclusion of additional costs of health care not related to the specific smoking-related diseases.

***Base analysis***

Table 2 summarizes the impact of an SFG policy after 10, 25, 50 and 90 years. The annual impacts tend to increase over time, although the annual number of disease cases prevented declines in later years, leading to a reduction in the annual health care costs avoided (Figures 2a, b and c). This results in a non-linear increase in cumulative impacts (Figures 3a, b and b).

**Figure 3A:**
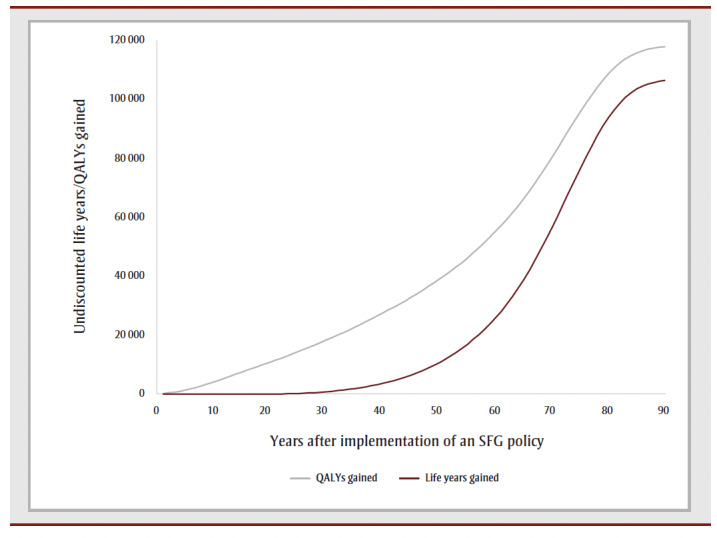
Annual undiscounted outcomes up to 90 Years—QALYs and life years gained

**Figure 3B:**
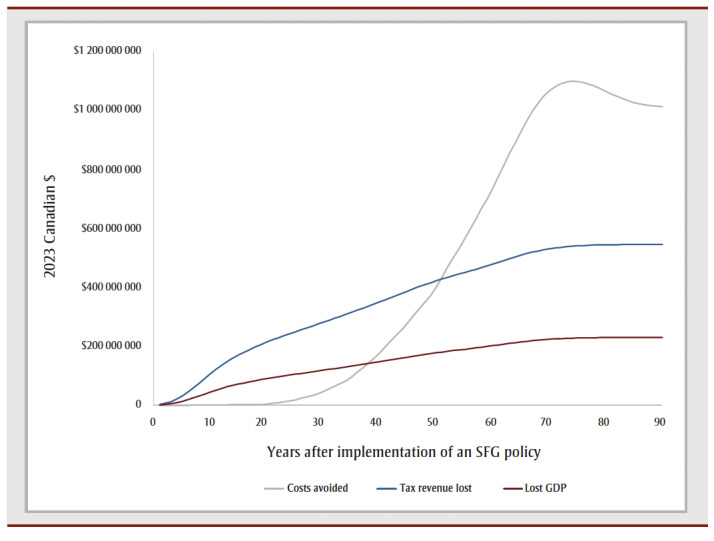
Annual undiscounted outcomes up to 90 years—financial gains and losses

**Figure 3C:**
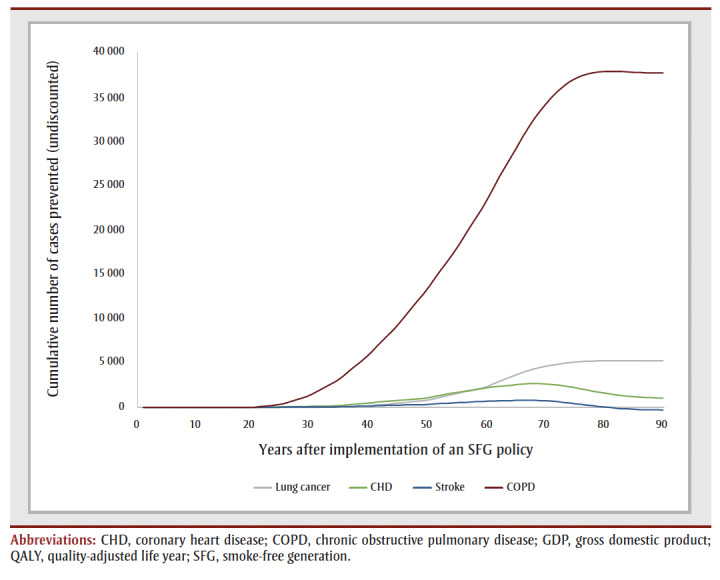
Annual undiscounted outcomes up to 90 years—cases prevented

After 10 years, the policy would not impact life years but would lead to substantive gains in QALYs (15700) and a modest decrease in health care costs for smoking-related diseases ($3.0million). The benefits increase over time with life years gained increasing to 214 after 25 years, 39475 after 50 years and 769042 after 90 years, QALYs gained increasing to 122766 after 25 years, 476814 after 50 years and 1545288 after 90 years and health care costs averted increasing to $66.7 million after 25 years, $2.3 billion after 50 years and $15.3 billion after 90 years. Similarly, the negative impacts of a policy on tobacco tax revenue and Canadian GDP from the tobacco industry increase over time: tax revenues foregone will be $403.5 million after 10 years, $2.6 billion after 25 years, $7.4 billion after 50 years and $14.7 billion after 90 years; decline in GDP would be $170.3 million after 10 years, $1.1 billion after 25 years, $3.1 billion after 50 years and $6.2 billion after 90 years (Table 2).

At 50 years, the proportion of outcomes that occur in females is 28% for life years, 35% for both QALYs and health care costs avoided, and 39% for both tax revenues foregone and decline in GDP (data not shown). Up until Year 9, the largest number of cumulative disease cases prevented is for lung cancer; from Year 10 until Year 21, the largest number of cumulative cases prevented is for CHD and thereafter the largest number of cumulative cases prevented is for COPD (Figure 3c).

For all years the cumulative health care costs averted are less than the sum of tax revenues foregone and decline in GDP, though after 58 years, the annual health care costs averted exceed the sum of tax revenues foregone and decline in GDP (Figures 2b and 3b). After 50 years, if a QALY was valued at at least $17147, the combined value of the QALYs gained and the health care costs averted would exceed the sum of lost tax revenues and reduction in GDP by $36423 after 10 years, $29738 after 25 years and $3605 after 90years (data not shown).

***Scenario analyses***

Scenario analyses for cumulative outcomes up to 50 years show that results are consistent across all scenarios explored (Table 4). Although the magnitude of impacts varies across scenarios, the relative values across each component remains consistent. Within the scenario analyses, the necessary threshold value for a QALY required for the SFG policy to be optimal, which was $17147 in the base case, varies between $14091 and $20909, highlighting the consistency in the results.

**Table 4:** Results of scenario analyses for cumulative outcomes up to 50 years

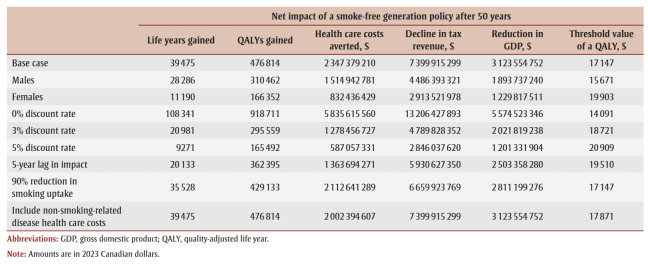

## Discussion

The results show that, based on the study assumptions, imposing an SFG policy will lead to substantive health benefits and reduced health care expenses that are only partially offset by reduced tax revenues from smoking and a decline in GDP. 

The general conclusions hold across multiple scenario analyses—including adding non–smoking-related disease health care costs, as scenario analysis found that when including all health care costs allowing for the increased life expectancy from the SFG policy, the SFG policy was still associated with reduced health care costs (a reduction of $2002394607 rather than $2347379210).

Analysis predicts that the government’s target for smoking prevalence of less than 5% would be achieved in 2035, should the SFG policy be introduced. Without the SFG policy, and based on current trends, the model predicts that a prevalence of less than 5% would be reached in 2040. The SFG policy would achieve further prevalence targets much more quickly. Of note, a smoking prevalence of less than 2.5% would be achieved in 2050 if the SFG policy were introduced but, without an SFG policy, this rate would not be reached until 2075.

Programs that reduce tobacco smoking also aim to reduce premature deaths, defined by Statistics Canada as deaths prior to age 75 years.^32^ The model predicts that for a cohort of 15-year-olds, premature deaths would be reduced from 20.2% without the SFG policy to 19.3% with the SFG policy, a relative reduction of 4.6%.

***Strengths and limitations***

A major strength of this study is that it uses data pertinent to the Canadian population. However, a number of caveats and limitations should be considered when reviewing the results.

Analysis included the impact of the imposition of an SFG policy on both the amount of tax revenues raised through tobacco sales and the reduction in GDP from the tobacco industry. A concern to do with the introduction of smoking cessation policies is the impact on tax revenue and government expenditure. The percentage reduction in overall tax revenue from an SFG policy is minimal when considered alongside total taxes and government expenditure: equivalent to only 0.2% of total income tax raised and 0.06% of total government expenditure. It is also worth noting that the current analysis does not consider the additional income tax raised by extensions to life expectancy through the SFG policy.

Further, decline in tax revenues is not a loss from a societal perspective as taxation is merely a transfer of funds from individuals to government. The impact of decreasing taxes will be uncertain as the changes in the level of taxation can be associated with either an increase or decrease in economic growth. Reducing the consumption of tobacco will lead to more disposable income available to consume other resources. Thus, by including both lost taxation and a decline in GDP as a negative offset, the estimated threshold values of a QALY required for an SFG policy to be beneficial are likely overestimated.

The analysis does not consider alternative policy options such as raising the legal age for the purchase of tobacco, access to vaping products, reducing nicotine standards for smoked tobacco products, increasing taxation on tobacco products or further restrictions on smoking in public.^33^ Such policies are not necessarily mutually exclusive when considering an SFG policy as many of these target current rather than potential smokers. Of note, an SFG policy is akin to raising the legal age for smoking by one year each year. Thus, it avoids the concern that by raising the legal age for smoking to a fixed age there is the high likelihood that those below the legal age will access tobacco products as the disparity between the legal age and the age at which smoking commences remains minimal.

Another limitation with the analysis is that it relies on data for some input parameters (e.g. smoking-related mortality, relapse) that are not recent. Analysis is based on the most contemporaneous data available, but if input parameters have changed noticeably, results may vary.

In addition, although input parameters for the uptake of smoking and quit rates for smoking are based on the most contemporaneous data, they may change over time. This analysis is based on the assumption that such rates will be stable over the time horizon of the model. If uptake rates were to decline without an SFG policy, then the benefits of the SFG policy would decline proportionally. However, the general conclusion is that the benefits are higher than the negative consequences would hold.

Another limitation with the analysis is that it does not consider all the potential benefits and costs of implementing an SFG policy. As noted, the analysis does not include any additional income tax raised by the increased life expectancy through the SFG policy. The analysis also does not consider the impact of an SFG policy on existing illegal markets for tobacco purchase or the costs of enforcing tobacco-related legislation (estimated at $37.6 million per year in 2012).^34^ Enforcement costs may grow in the initial years of implementation of an SFG policy but could subsequently decline as the prevalence of smoking declines across the population.

The arguments against tobacco control policies such as an SFG policy from advocates for the tobacco industry tend to focus on three areas: the denial of freedom of behaviour; the unworkability of such a policy; and the contribution of the tobacco industry to society through both GDP and tax generation.^6^ It could be argued that the first argument is the least addressed by the analysis in this study. However, it is important to note that tobacco addiction is often initiated during childhood or at least before the age of 21years, the minimum age for selling tobacco in many provinces. Further, the analysis does not incorporate any additional health and health care benefits from the decline in passive smoking as a result of an SFG policy. Analysis does demonstrate the substantive benefits of an SFG policy even if there is a lag in its effects or a less than perfect cessation of smoking uptake. Thus, concerns relating to unworkability are irrelevant. Analysis directly addresses the final argument by not focusing solely on the impacts on health and health care consumption.

## Conclusion

This study highlights the impacts of imposing an SFG policy across Canada, and demonstrates that the health benefits and reduction in health care costs outweigh any positive contribution of tobacco smoking to the Canadian population.

## Acknowledgements

Analysis is based on a revised version of a model developed in partnership with Dr. Catherine Pound of the University of Ottawa, Ottawa, Ontario, Canada.

## Funding

None.

## Conflicts of interest

None.

## Authors’ contributions and statement

DC: Conceptualization, methodology, formal analysis, writing – original draft, writing – review & editing.

The content and views expressed in this article are those of the author and do not necessarily reflect those of the Government of Canada.
